# The role of early immunotherapy in rasmussen’s encephalitis

**DOI:** 10.1007/s10072-025-08711-9

**Published:** 2026-01-17

**Authors:** Eric J. Ronne, Mellad M. Khoshnood, Jonathan D. Santoro

**Affiliations:** 1 Division of Neurology, Department of Pediatrics, University of California Los Angeles, Los Angeles, CA USA; 2 Cottage Hospital, Santa Barbara, CA USA; 3 Division of Neurology, Department of Pediatrics, Children’s Hospital Los Angeles, 4650 Sunset Blvd, MS82, Los Angeles, CA USA

**Keywords:** Rasmussen's encephalitis, Epilepsia partialis continua, Immunotherapy, Epilepsy, Hemisphere

## Abstract

Rasmussen’s Encephalitis (RE) is a rare clinical syndrome of chronic progressive unihemispheric cortical inflammation and atrophy, with explosive focal onset seizures which are commonly intractable and may include epilepsia partialis continua (EPC), unilateral neurologic deficits, and cognitive decline. Despite awareness of this disease since characterization in 1958, the underlying pathogenesis of RE remains unknown. Neurosurgical intervention, via functional hemispherectomy of the affected cerebral hemisphere, remains the standard of care for RE. However, due to the invasive nature of this procedure, and emerging evidence for a neuroinflammatory pathogenesis, various immunologic mechanisms have been proposed and immunotherapies trialed for patients with RE. The rare nature of the condition and heterogenous data in the literature have made determination of the role of immunotherapies, such as rituximab, difficult to ascertain. Here we report a series of three patients with pediatric RE, all of whom received early administration of immunotherapy in the context of restricted oligoclonal bands in the cerebrospinal fluid. While two patients were non-responders, one patient exhibited no radiographic progression on rituximab and one of those patients had clinical improvement. We discuss the potential role and mechanism of rituximab as well as the proposed mechanisms and available data for immunotherapy in RE more broadly. Although data remains limited, biomarkers of immunologic dysregulation may serve as indications for the use of immunotherapy in individuals with RE. Further, selection of immunotherapy, guided by ancillary testing including CSF analysis, may serve as an adjunct to hemispherectomy and neurosurgical intervention in some patients.

## Introduction

**Fig. 1:**
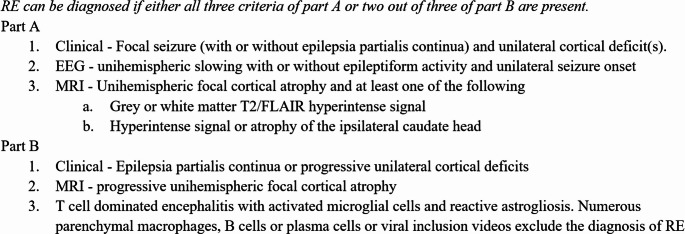
Bien criteria for diagnosis of Rasmussen’s Encephalitis [4]

Rasmussen’s Encephalitis (RE), first characterized by neurosurgeon and epilepsy surgery pioneer Theodore Rasmussen in 1958, is an ultra-rare clinical syndrome of chronic progressive unihemispheric cortical atrophy, with explosive focal onset seizures which are commonly intractable and may include epilepsia partialis continua (EPC), unilateral neurologic deficits, and cognitive decline [1–4]. This condition is presumed to affect 2.4 individuals per 10 million [3]. Figure 1 provides the current diagnostic criteria for RE, originally proposed by Bien et al., in 2005 [4].

Despite awareness of this condition, a definitive pathophysiology remains elusive, complicating the selection of non-surgical interventions. Although utilization of immunotherapy is increasingly reported in the literature, standardized approaches and timing of initiation have remained heterogeneous [5]. In the era of targeted therapies and monoclonal antibodies, there is a significant need to re-evaluate well established diseases without definitive treatment pathways. This is pertinent for RE, wherein hemispherectomy carries both immediate and long-term risks for neurological sequelae [6]. Here, we present a case series of children with RE who received early immunotherapy. We discuss the current literature and the emerging evidence for immunotherapeutic treatments of RE, with an emphasis on the potential role of early medical management with immunomodulation, such as rituximab.

## Methods

### Regulatory approval and data availability

This retrospective chart-based study was approved by the Children’s Hospital Los Angeles Institutional Review Board (CHLA-20-00004). Given the retrospective nature of the study, consent and assent were waived. Anonymized data is available to qualified researchers upon IRB approval.

## Case data extraction

Patients with RE were identified through the course of practice by the authors. Demographic, radiographic, and clinical data, as well as treatment protocols were extracted from the electronic medical record by the authors (EJR MMK).

## Literature review and search strategy

The authors conducted a literature review to identify appropriate studies and searched the PubMed, Web of Science, and Scopus databases on February 29, 2024, and again on December 15th, 2024, for publications describing treatment of RE with second line immunotherapeutics. No restrictions on the date of publication were applied. The authors included manuscripts published in English only. Manuscripts were reviewed at the abstract level for inclusion if they pertained to RE. Following abstract identification, authors reviewed each manuscript in full. For inclusion in this study, articles had to (1) include individuals diagnosed with RE based on Bien criteria [4] and (2) report treatment with any form of second-line immunotherapy. Second-line immunotherapy was defined as intravenous immunoglobulin (IVIg), plasmapheresis, tocilizumab, azathioprine, mycophenolate, tacrolimus, natalizumab, cyclophosphamide, anakinra, hematopoietic stem cell transplantation, methotrexate, basilixumab, adalimumab or rituximab. Treatments in broad categories that were selected for review a priori (B-cell/Antibody mediated therapies, T-cell therapies, and microglial therapy classes) were also included regardless of evidence level. Case series were included for review although individual case reports which were singular in the literature (e.g., there was only one case report of use) were excluded, with the exception of when multiple single case reports were available for a particular immunotherapeutic. Manuscripts not meeting these criteria were excluded. Manuscripts partially meeting these criteria were reviewed by all authors and adjudicated based on relevance.

## Case series

Three children diagnosed with RE were identified through the course of practice at an academic, quaternary care medical center in the United States. A summary of their clinical courses and neurodiagnostic studies are available in Table 1. Relevant CSF findings are noted in Table 2. Cases 1 and 2 follow a traditional clinical course and treatment for RE, wherein these patients presented with explosive onset seizures, associated hemiatrophy on MRI, and progressive hemiparesis on exam. These patients ultimately proceeded to functional hemispherectomy despite early and aggressive immunotherapy as detailed below. Case 3 represents an atypical presentation years after seizure onset who also achieved radiographic and clinical stabilization after immunotherapy, and even reversal of some neurological deficits. Additional clinical information for each case may be referenced in narrative form in the discussion below.

**Table 1:** Clinical course (disease duration before treatment in other studies of Immunologic therapy)

	Case 1	Case 2	Case 3
Demographics	5y RH M, history of autism	5y RH F with no past medical history	13y RH M with no past medical history
Onset of epilepsy	3y 7 m	3y 6 m	3y 9 m
Epilepsia Partialis Continua	No	Yes, left leg twitching	No
Seizure Burden at Diagnosis	> 25 seizures per day	> 20 seizures per day	5 seizures per day
Initial EEG findings	Left temporal electroclinical seizures. Left temporal interictal epileptiform discharges and runs without clinical correlate. Left temporal slowing and L hemisphere attenuation.	Persistent, focal delta slowing over the right (maximal parasagittal and posterior region). Rare right parasagittal epileptiform discharges	Frequent sharps/sharp wave complexes of high amplitude over right vertex and central regions, near continuous in sleep. Focal slowing over right central and vertex regions.
Failed Anti-Seizure Medications	Phenobarbital Zonisamide Levetiracetam Valproic Acid	Oxcarbazepine Clobazam Zonisamide	Levetiracetam Lacosamide Oxcarbazepine Valproic Acid Topiramate Clobazam
Anti-Seizure Medications at Time of Immunotherapy	Clobazam Lacosamide Perampanel Phenytoin Clonazepam	Levetiracetam Lacosamide	Oxcarbazepine Cannabidiol Clonazepam PRN Vagus Nerve Stimulation (VNS)
Initial MRI	L frontal lobe, postcentral gyrus, and insula gliosis and volume loss, associated ex vacuo dilatation of ventricles L mesial temporal sclerosis	Mild diffuse volume loss of the R cerebral hemisphere Scattered increased T2 signal within the cortical and subcortical white matter of the R cerebral hemisphere and R insula	Subtle white matter changes in the R frontal lobe, extending from the lateral ventricle. Questionable cortical abnormalities. No other abnormalities w/and w/out contrast.
PET-FDG CT	Decreased uptake in L frontal and L temporal lobes, no areas of increased uptake	Not obtained	3 years from onset: Increased FDG uptake along posterior L frontal lobe adjacent to falx presumably distorted attenuation correction
Genetic testing	*PIGS* Heterozygous c.398delA p.Glu133Glys*10 (likely pathogenic) *PSAP* (details unavailable, classified VUS) *TBL1XR1* (details unavailable, classified VUS)	*PIGQ* c.223G > T (p.Glu75*) (pathogenic) *SNIP1* c583G > A (p.Val195lle) (VUS) *SPTAN1* c.2648G > A (p.Arg883Gln) (VUS)	*CIC* Heterozygous (VUS) c.4144G > A, p.Asp1382Asn *CEP85L* Heterozygous (VUS) c.1762G > A, p.Glu588Lys
Neuropsychologic testing	Yes: 12 weeks from disease onset; average receptive vocabulary, below average expressive vocabulary, exceptionally low adaptive skills (as rated by mother), and multiple concerns regarding emotional and behavioral functioning	Yes, 20 weeks after disease onset; low average overall intellectual functioning, with a weakness in visual-spatial working memory	None
Immune therapies (time from seizure onset)	IVMP (9 weeks from onset) IVIg (10 weeks from onset) Rituximab (11 weeks from onset)	IVMP (8 weeks after onset) IVIg (8 weeks after onset) Rituximab (10 weeks after onset)	IVIg (4 years from onset) IVMP (4 years from onset) Rituximab (8 years from onset, 9 years from onset) Tocilizumab (10 years from onset)
Disease stabilization for > 6 months	No Equivocal response to IVIG and rituximab	No Transient response to IVMP	Yes Radiographic stability on Rituximab Clinical improvement on Tocilizumab
Surgical Intervention	Yes 5 months after onset	Yes 16 months after onset	No

**Table 2:** CSF findings in cases

	Case 1 (9 weeks from onset)	Case 2 (8 weeks from onset)	Case 3 (6 years from onset)
WBC (cell/mm3) *Range < 5 cells/mm3*	3	2	**7**
Protein (mg/dL) *Range 10–45 mg/dL*	37	15	18
Glucose (mg/dL) *Range 33–70 mg/dL*	52	49	57
IgG synthesis rate *Range < 3.3 mg/day*	−1.2	**18.4**	−0.7
IgG index *Range < 0.70*	0.63	**3.71**	0.63
Oligoclonal bands *Range: none*	**> 5 restricted**	**> 5 restricted**	**> 5 restricted**
Neopterin (RR 7–65) *Range: 7–65 nmol/L*	**224**	**206**	14
Neurotransmitters	Normal	Not obtained	Normal
Cytokines	Not obtained	Normal CSF cytokines	**IL-6: 34 pg/mL (normal < 7.5pg/mL)**

Legend: bold items are abnormal

In Cases 1 and 2, IVMP and IVIg were trialed within two weeks of presentation and then followed with next-line immunotherapy due to incomplete or transient response. In Case 1, CSF demonstrated > 5 restricted oligoclonal bands and in Case 2, > 5 restricted oligoclonal bands and elevated IgG synthesis rate and IgG index were present. Rituximab was selected as the next-line immunotherapy and started within 12 weeks of presentation for each. Despite this, Case 1 and Case 2 ultimately continued to have refractory epilepsy and proceeded to functional hemispherectomy at five months after disease onset and 16 months after disease onset, respectively.

### Case 3

is unique in that they had a slow and insidious left hemiparesis, with right hemispheric atrophy and signal changes on MRI over a period of six years, leading to a diagnosis of RE on the basis of the above findings, unilaterality of disease, and a broad but otherwise unrevealing workup for alternative etiologies. The MRI findings stabilized following initiation of rituximab (selected in part due to presence of restricted oligoclonal bands) and subsequent use of tocilizumab (selected due to elevated IL-6 on CSF cytokine panel) led to marked clinical improvements as well, including improvement in speech and language. While the patient has regained some limited expressive speech and are now able to speak in 2–3-word sentences. Further, seizure burden has reduced considerably having decreased by 75% from peak symptoms.

In all cases, immunotherapy was well tolerated. There were no acute infusion reactions in any patients although insomnia, irritability and agitation were noted in all patients who received IVMP. No patient had atypical infections, lymphopenia, leukopenia, neutropenia, or hypogammaglobulinemia at last follow-up.

### The potential role of immunotherapy in rasmussen’s encephalitis

## Results

### Inclusion of manuscripts for detailing of immunotherapy in RE

In total, 46 manuscripts were identified for abstract level review. Of these, 21 (46%) were reviewed in full. Following adjudication, 16 (35%) were included. There were no manuscripts which required adjudication during the review process.

## Immunologic mechanisms and Understanding

**Fig. 2:**
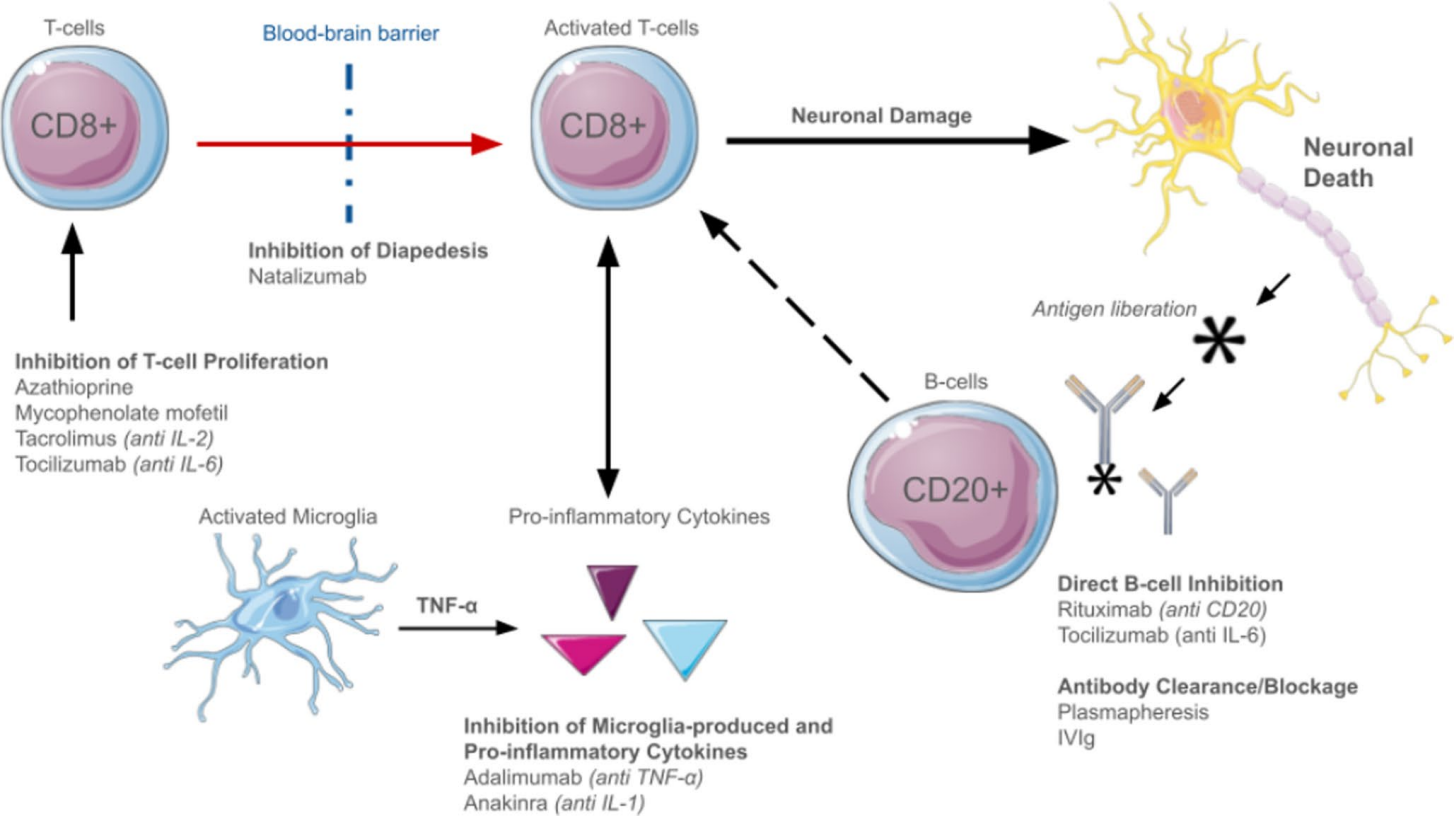
Immunologic mechanisms implicated in the pathophysiology of RE and associated immunotherapy treatment targets (Illustrations from smart.servier.com)

Proposed mechanisms underlying RE and its treatments are detailed in Fig. 2; Table 3, respectively. Multiple immunologic mechanisms involved in RE have been reported, however, the precise underlying pathogenesis of RE remains poorly understood. T-cell and microglial activation have been demonstrated on biopsy specimens and concurrent findings suggesting co-activation of the humoral immune system have been seen [10]. Here we review three broad pathophysiologic categories (antibody-mediated, t-cell cytotoxic, and microglia-induced inflammation) and identify the immunotherapies that may be employed based on each. Please note that the discussion of second-line immunotherapies is limited to those with the widest usage and evidence base. A plethora of immunotherapies have been trialed in RE in isolated case reports or short series, such as anakinra, cyclophosphamide, mycophenolate mofetil, natalizumab, and others [11].

**Table 3:** Possible Immunologic mechanisms in RE

	Antibody-mediated	T-cell Cytotoxicity	Microglia-induced
Rationale	Multiple neuroinflammatory disorders are associated with circulating or CSF-restricted antibodies to neuronal surface proteins. Such autoantibodies, as well as restricted oligoclonal bands, have often been identified in cases of RE [5, 7].	Significant T-cell infiltration is seen on biopsy specimens. Spectra-typing shows expansion from discrete epitope-responding precursor T-cells [8, 9].	Microglia activation is a pathological hallmark of RE which follows the pattern of T-cell infiltration. Resultant cytokine release is suspected to contribute to seizure induction [5, 8].
Molecular Target(s)	CD20, direct B-cell inhibition, reduction of antibody production.	Calcineurin, inhibiting IL-2 transcription and lymphocyte signaling, inhibition of IL-6.	anti-TNF-α, inhibiting microglial activation
Potential Treatment(s)	Rituximab Plasmapheresis *IVIg	Tacrolimus Azathioprine Tocilizumab	Adalimumab

### Antibody mediated inflammatory response

Autoantibodies (such as anti-GluR3, Munc18-1, AMPAR, α7 nAChR) have been identified in the serum of RE patients, however the role of B-cells and autoantibodies in the pathogenesis of RE remains unclear [7, 12–14]. Many other neuroinflammatory disorders are known to involve either a discrete autoantibody or CSF-restricted antibodies, such as those found in individuals with autoimmune encephalitis caused by anti-NMDA receptor autoantibodies [15]. Testing for discrete antibodies such as these in the CSF is important for individuals with suspected RE although other markers of B-cell activity such as the presence of oligoclonal bands, IgG index elevation and/or B-cell population expansion on cytology can be used to determine non-specific immunologic dysregulation [16]. While activation of the humoral immune system may simply occur alongside the primary T-cell or microglial-mediated process in RE, its modulation may still be able to impact the inflammatory cascade through a secondary effect of attenuating interdependent T-cell activity [17]. From a treatment standpoint, the anti-CD20 therapy rituximab has been utilized as a second-line immunotherapy on this basis [18, 19]. Plasmapheresis clears antibodies from the serum and therefore attenuates antibody-mediated inflammation. It is worth noting that IVIg may also provide antibody-mediated benefits although the mechanism of action is broad [20].

### T-Cell dependent cytotoxicity

The basis for T-cell mediated cytotoxicity in RE is more clearly established in the literature. Brain biopsy specimens in patients with RE have demonstrated T-cell infiltrates, with cytotoxic CD3 + and particularly CD8 + T-lymphocytes predominantly seen [21]. Significant infiltration of resident memory T cells (CD8+, CD103+) is additionally noted as well as clonal expansion of T-cell populations beginning early on in the disease course. This suggests a T-cell response to a specific, yet-to-be identified, epitope(s) in the CNS [22]. These reports indicate a broader T-cell over-activation state but have not appreciably demonstrated a consistent pathophysiologic pathway in RE.

T-cell overactivation is targeted by multiple therapeutics but in RE. Tacrolimus, which inhibits CD8 + T-lymphocyte proliferation and activation via inhibition of IL-2 gene transcription, has been used frequently. Azathioprine similarly exerts an anti-T-cell effect via inhibition of purine synthesis, inhibiting T-cell proliferation and leading to apoptosis of activated T-cells. Additionally, IL-6 is another potential therapeutic target, as it drives cytotoxic T-cell differentiation and may be competitively inhibited at its receptor by the monoclonal antibody tocilizumab [23]. IL-6 is also involved in stimulation of B-cells and antibody production [23]. Therefore, while not a specific T-lymphocyte-targeted therapy, tocilizumab may provide benefit through blockade of downstream pathways.

### Microglial activation

Alongside T-cell infiltration, microglial activation is reported on histological brain specimens in patients with RE. Through production and release of pro-inflammatory cytokines, activated microglia are proposed to contribute to both ictogenesis and ongoing inflammatory response through further recruitment and activation of T-cells [5]. Activated microglia exert many immunomodulatory effects through transcription and release of TNF-α which has pleiotropic effects on several different cell types and is known to be involved in the pathogenesis of several inflammatory disorders. Microglia are some of the most interconnected cell types in the CNS, receiving and sending a wide variety of signals from adjacent tissue and as such may play a central role in the RE disease process [24].

TNF-α is directly bound and inactivated by monoclonal antibody Adalimumab, thereby limiting the pro-inflammatory effects of activated microglia and preventing further microglial activation [8, 25]. It should be noted that there is some suspicion that TNF-alpha also has an immune regulating effect, and thus its blocking can result in the development of autoimmune diseases such as acquired demyelinating disorders [25].

### Genetics and rasmussen’s encephalitis: A new frontier for targeted therapies

The landscape of genetic testing and disease identification is expanding considerably. Unfortunately, this has manifested in the form of many variants of uncertain significance (VUS) due to genetic variability amongst populations not previously studied [11]. Assessment of the clinical significance of these VUS remains limited and must be undertaken on a patient-by-patient basis. As such RE may involve underlying genetic predisposition to the disease process although we cannot reasonably come to any conclusions at this time. This is especially so given the heterogeneity of VUS identified amongst our patients. That said, we will also note the benefit of genetic testing in identification of disorders of immune dysregulation in neurologic diseases, potentially leading to more precisely targeted therapies in the future [26]. The greatest benefit of rapid genetic testing includes identification of mitochondrial disorders, which are a known cause of EPC and can be exacerbated by some immunotherapies, particularly steroids [27]. As such, genetic testing should be part of diagnostic testing in RE.

### Immunotherapeutic data in rasmussen’s encephalitis

Available data for immunotherapies is detailed in Table 4.

**Table 4:** Available data for specific immunotherapies by class

	B-cell/Antibody mediated	T-cell therapies		Microglial
	Rituximab	Tacrolimus	Azathioprine	Adalimumab
Available data	9 patient case series [19]. 4 patient case series (This paper) Isolated case reports [18, 28–32]	7 and 9 patient case series with comparison to historical cohorts [3, 4]. 12 patient case series [33]. Isolated case reports [31].	30 patient retrospective study [34]. Isolated case reports [35–38].	Open label study of 11 patients [35].
Findings	Some efficacy in reducing seizure burden, few cases in which clinical progression of disease was arrested	Moderate evidence for slowing of clinical decline but does not reduce seizure burden	Some effectiveness for seizures but otherwise minimal impact on disease course	5 of 11 patients classified as responders, with > 50% reduction in seizure frequency, 3 of those 5 patients with clinical stabilization as well
Other Therapies	Treatments with lower volume of reports which were not included in this analysis include IVIg, mycophenolate mofetil, cyclophosphamide, methotrexate (systemic and intrathecal), nataliuzmab, basilixuimab, alemtuzumab, anakinra, and hematopoietic stem cell transplantation.			

## Discussion

RE is a complex and possibly polyfactorial disease with significant variability in both initial presentation and in ultimate clinical course. Immunotherapy has the potential to modify the disease course in RE, and in a subset of patients may fully arrest progression and avoid the need for hemispherectomy.

One point to be made with regard to all cases is the potential role that timing may play in the success or failure of immunotherapy. In Cases 1 and 2, rituximab was initiated 11 and 10 weeks from disease onset, which represents a shift in treatment approach from earlier cases and case series where immunotherapy was often trialed much further into the course of RE, often ranging from several months to years after disease onset [8]. Like many autoinflammatory diseases of the nervous system, first line therapies like IVMP and IVIg see variable response, although there is data to suggest a time-sensitive response to IVIg in RE with better outcomes for earlier treatment [39]. Second-line immunotherapies and earlier treatments have been shown to be important in the autoimmune encephalitis literature, with recent data suggesting that treatment with second line therapies beginning < 3 months from disease onset may yield a more favorable long-term outcome [40, 41]. This is additionally corroborated by recent data in RE, with a robust response seen in a pediatric patient who received rituximab within two months of disease onset [19].

Phenotypic variance is another key feature of RE, with significant variance in both age of onset and aggressiveness of disease course [42]. In contrast to Case 1 and Case 2, who presented at 5 years of age with rapidly progressive disease, ultimately proceeding to hemispherectomy after lack of response to immunotherapy. Conversely Case was an immunotherapy responder. Case 3, presenting at similar age to Cases 1 and 2, had a more chronic course, had notable reduction in seizure burden with rituximab and a robust clinical and radiographic response to tocilizumab. This could suggest that age of onset and/or pre-treatment course could prove to be predictors of success in usage of second line therapies. That said, there is an overall paucity of data as it pertains to the granular distinctions of age of onset in pediatric patients, and more research is necessary to draw any generalizable conclusions.

In this study, two patients had potentially pathogenic gene mutations that are associated with epilepsy. Patient one (*PIGS*, c.398delA p.Glu133Glys*) had a heterozygous gene mutation which was presumed to be pathogenic based on the molecular deletion of the phosphatidylinositol glycan anchor biosynthesis class S protein which could cause seizure/epilepsy. That said, there is essentially no literature on the *PIGS* gene available. Patient two’s gene mutation (*PIGQ* c.223G > T (p.Glu75*) is associated with early-onset epilepsy, severe developmental delay and elevations in serum alkaline phosphatase. The patient’s phenotype did not match the established phenotype in the medical literature. Neither the *PIGS* nor *PIGO* genes are associated with immune dysregulation. The genetic abnormalities noted in this study are interesting in that they could either act as a nidus for the development of RE, conversely, be the primary cause for the patient’s epilepsy. Further research into the genetics of RE is warranted as generalization is not possible using this small data set.

Unfortunately, no comparative data exist for second-line immunotherapies due to the inherent challenges of studying a rare disease without a standardized immunotherapy treatment algorithm. Viewed as a whole, there is no clear pathophysiologic evidence to support the superiority of any specific immunotherapy in RE. Immunotherapy therefore exists alongside definitive treatment in the form of surgery. Early consideration of immunotherapy in cases or RE is reasonable as literature of other neuroinflammatory diseases such as multiple sclerosis and autoimmune encephalitis seem to benefit from early initiation [43, 44]. Notably, the use of immunotherapy does not appear to lead to delay in surgery or adverse modification of post-surgical outcomes [6, 9]. This is critical, as hemispherectomy outcome data suggest that surgery should be performed as early as possible, highlighting the long-standing concern that immunotherapy may be a disservice if it merely slows decline and postpones definitive surgical treatment. Whether immunotherapy could augment surgical outcomes remains unknown, but is a potential area of interest for future research.

In the absence of definitive recommendations or data from the literature, we recommend utilization of ancillary immunologic findings to guide consideration of second-line immunotherapy should first-line options (IVMP, IVIg) fail to be efficacious. Rituximab is a rational and well-tolerated selection if evidence of an antibody or B-cell-mediated process is present. In the setting of elevated neopterin or otherwise non-specific findings, T-cell mediated therapies are reasonable to consider, though lack of consistent efficacy from T-cell targeting therapies must be noted. There is merit in acquisition of CSF cytokines, which may be helpful in selecting further immunotherapies, as with tocilizumab in Case 3. Immunotherapy should always be employed in addition to the initiation of a surgical evaluation, and the risk/benefit profile must be weighed by the treating clinician. Even if a case ultimately progresses to surgery, there is value in controlling seizures and potentially slowing MRI changes and functional decline in the pre-surgical period. There may be a subset of patients who respond robustly to immunotherapy and have the ability to be maintained on long term medical treatment alone or proceed to a more localized resection targeting an epileptogenic focus as opposed to full hemispherectomy. This treatment paradigm is not dissimilar to what is employed in neuro-oncology fields at present where targeted medical therapies which reduce tumor burden optimize surgical and functional outcomes [45].

This series is not without limitation. The study is limited by its retrospective design, low patient counts, and heterogeneity between cases. This study was performed at a quaternary academic medical center which may have skewed cases to be more medically severe, limiting the generalizability and skewing towards more severe outcomes. Further, our center utilizes a center-specific practice of early and aggressive immunotherapy in children with potential neuroinflammatory disorders which biases the data towards treatment. This study’s literature review was limited by the existing base of data for usage of immunotherapies in RE, which was further compounded by the retrospective designs and significant variability in treatment pathways, timing of administration, and order of immunotherapy. These factors appear to be important in determining success or failure of immunotherapy and therefore this heterogeneity may severely limit our ability to draw conclusions from existing literature. Some data comes from adult patients with RE which may further limit generalizability to the typical pediatric-onset form. Additionally, no comparative data exists between immunotherapies and hemispherectomy, the current standard of care for RE. Finally, although not a focus of this review, the importance of timing for immunotherapy highlights one limitation of Bien criteria for RE diagnosis, which necessitates characteristic MRI findings be present at the time of diagnosis. If characteristic findings are not yet present due to capture of a patient very early in the disease course, the diagnostic criteria will not be met. This places a limitation on speed with which diagnosis and treatment (whether immunotherapeutic or surgical) can be pursued.

Emerging data continues to change our understanding of RE although therapeutic studies continue to be limited in the setting of a complex and heterogeneous disease. Development of multi-center retrospective and prospective therapeutic studies are needed in addition to long-term follow data for immunotherapy-treated RE. In the current practice environment, use of advanced diagnostic testing can help guide immunotherapy choice when warranted.

### Rituximab

Rituximab has become a mainstay therapy for autoimmune encephalitis and other antibody mediated neurologic diseases [46]. It has been shown that early use of rituximab for anti-NMDA-receptor encephalitis, even in those responsive to first line therapies, has led to an improvement in outcomes and a reduction in relapse rate [40]. Rituximab is well-tolerated amongst pediatric patients, with a largely manageable side effect profile [46]. Data on the use of rituximab in RE has been limited, composed of isolated case reports that indicate some effect on seizure burden primarily, and clinical improvement in one adult patient [18]. In a 2022 study, where nine patients with RE received rituximab, patients who received early intervention (< 1 month) had no progression of disease and obtained seizure freedom. Conversely, only some of those receiving treatment further into their RE course achieved slowing of disease progression and improvement in seizure burden [19]. No significant adverse events were reported. These data suggest that there may active B-cell/autoantibody driven pathophysiology during early phases of RE, which could be modifiable.

### Tacrolimus and azathioprine

Tacrolimus has been studied in RE across three separate case series and various case reports. The data taken together indicate no significant effect on seizure burden although one study observed a response rate of 42% [33]. The two Bien series revealed tacrolimus slowed both MRI atrophy and clinical exam deterioration as compared to a historical cohort [2, 21]. Additionally, 75% of the patients in Takahashi et al. 2013 had cognitive stabilization with 1 of the 12 patients experiencing motor improvement [33]. Of note, two patients receiving tacrolimus in the Bien 2013 series did suffer severe infections as a consequence of immunotherapy [3].

Where tacrolimus seems to have minimal effectiveness for seizure control but some effect in slowing clinical deterioration, the reverse seems to be true for azathioprine. One retrospective study reported good efficacy for seizures with response to azathioprine in 25 of 30 patients (83%), but no clear effect on other clinical symptoms or progression of MRI findings [34]. There may therefore be a niche in specific cases that present primarily with clinical and MRI findings, or primarily with seizures, for use of tacrolimus or azathioprine respectively.

### Tocilizumab

To our knowledge, Case 3 is the first example of a presumed RE case treated with tocilizumab. The acute improvements in seizure burden and clinical symptoms after initiation were marked, even despite the patient’s long-standing disease. CSF cytokines could prove a useful biomarker; however, IL-6 elevation is unlikely to be specific to RE and may in fact be more suggestive of a broader immunologic process. IL-6 is notable as a pro- and anti-inflammatory cytokine upregulating T and B cell activity [47]. Given the association of RE pathophysiology with these lineages, it could prove a useful target if more data can be obtained suggesting a correlation. It is therefore sensible to obtain CSF cytokines as part of routine RE workup to determine if tocilizumab may be an appropriate second- or third-line immunotherapy. As with other immune therapies, Tocilizumab has reasonable data from other populations to conclude good tolerance in pediatric patients [48].

### Adalimumab

Adalimumab is an anti-TNF-alpha monoclonal antibody that effectively disrupts the pro-inflammatory effects of TNF-alpha leading to systemic immunosuppression and regulation [25]. A case series of eleven pediatric patients who received adalimumab with a median delay of 31 months from disease onset showed a reduction of seizure burden by at least 50% in 5 of 11 patients (45%) and improvements in motor weakness and cognitive function in 3 of those 5 patients (60%). Although not widely studied, the limited data available may support the use of adalimumab in slowly progressive forms of RE.

### Surgical outcomes

Hemispherectomy is the only well-established intervention for RE, and therefore surgical outcomes are the reference to which other interventions must be compared. Despite the invasiveness of this procedure, hemispherectomy remains well tolerated and long-term seizure outcomes are excellent [49]. In a 30-patient retrospective study in 2022, post-surgical seizure freedom rate was 81.5%, 63.6%, and 55.6% at 1, 5, and 10 years, respectively [50]. This is corroborated by a 2024 study including 44 patients with seizure freedom at latest follow up in 68% [51]. Regarding motor/functional, and cognitive outcomes, all 11 patients in a series in 2016 were ambulatory at follow up [6]. A 2024 study involving 44 patients found that preoperative functional status is often preserved, even when the left hemisphere is involved (64% with stable postoperative cognitive function, 54% with stable gross motor function, 74% with functional hand use) [51]. Language is of particular interest in RE as roughly half of cases will involve the dominant hemisphere. In a 2022 series of 40 patients, cognitive dysfunction was found to improve post-surgery, however non-dominant hemispherectomies achieved better language and verbal IQ outcomes [52]. A shorter series in 2015 demonstrated the potential for complete functional reorganization of language with recovery to a normal verbal IQ (> 70) in all six patients [53]. The literature supports the argument that earlier hemispherectomies achieve better outcomes, particularly in cognitive and language domains, through earlier remapping of cognitive functions to the unaffected hemisphere [52, 54–56]. Given strong data for seizure response and acceptable functional, cognitive, and language outcomes, surgical treatment remains the gold standard intervention for RE. Immunotherapy therefore exists as an adjunct treatment in the presurgical period or primary treatment in cases where surgery may not be possible.

## Abbreviations

RE: Rasmussen’s Encephalitis
EEG: electroencephalography
MRI: magnetic resonance imaging
PET-FDG: 18 F-fluorodeoxyglucose positron-emission tomography
CSF: cerebrospinal fluid
CNS: central nervous system
ASM: anti-seizure medications
IFN: interferon
IL: interleukin
TNF: tumor necrosis factor
EPC: epilepsia partialis continua
IVMP: intravenous methylprednisolone
IVIg: intravenous immunoglobulins
